# Familial aggregation of components of the multiple metabolic syndrome in the Framingham Heart and Offspring Cohorts: Genetic Analysis Workshop Problem 1

**DOI:** 10.1186/1471-2156-4-S1-S94

**Published:** 2003-12-31

**Authors:** Kristine E Lee, Barbara EK Klein, Ronald Klein

**Affiliations:** 1 Department of Ophthalmology and Visual Sciences, University of Wisconsin Medical School, Madison, Wisconsin, USA

## Abstract

**Background:**

The multiple metabolic syndrome is defined by a clustering of risk factors for cardiovascular disease. We sought to evaluate the familial correlations of the components of the syndrome using data from the Framingham Heart Study original and offspring cohorts as provided for the Genetic Analysis Workshop 13. Measures of plasma cholesterol (total and HDL), body mass index (BMI), and systolic blood pressure were used from selected calendar years of exams. Familial correlations were calculated using FCOR in S.A.G.E.

**Results:**

The sibling correlations were relatively high for all measures and exams, from 0.17 for systolic blood pressure to 0.27 for HDL cholesterol. The parent-child correlations were very similar, except for systolic blood pressure. The avuncular correlations were much smaller and the cousin correlations were even smaller. For HDL cholesterol the avuncular correlation was half the sibling correlation and the cousin correlation was half that again. Spousal correlations ranged from 0.07 for systolic blood pressure to 0.34 for BMI. Correlations were somewhat lower from 1984 to 1987 examinations than from 1971 to 1975 examinations, except for spousal correlations for systolic blood pressure and BMI.

**Conclusion:**

The results of the family pair correlations are suggestive of genetic determinants of lipid levels and BMI. These components have been shown to be predictive of cardiovascular disease as well as diabetes. Genes in common with each of the components might also influence development of cardiovascular disease and diabetes, both complex diseases.

## Background

The multiple metabolic syndrome, also known as syndrome X or the insulin resistance syndrome, is defined by a clustering of risk factors for cardiovascular disease. Although there is no common definition of the syndrome, it usually includes high plasma triglycerides, low HDL cholesterol, glucose intolerance, high blood pressure, obesity, and proteinuria [1]. Persons with the syndrome have increased risks of developing diabetes as well as cardiovascular disease [2,3]. The risks of disease are greater for those with the syndrome than for each of the risk factors separately.

Some studies have begun to look at familial relationships of the syndrome. First-degree relatives of persons with diabetes are more likely to have the syndrome [4,5]. Persons with the syndrome are more likely to have family members with the components [6,7]. Models suggest that the same set of genes is involved with each component [8,9]. We used the Framingham Heart Study original and offspring data provided for the Genetic Analysis Workshop 13 to investigate the familial relationships of the components of the multiple metabolic syndrome.

## Methods

Data have been provided from the Framingham Heart study original and offspring cohorts. The Framingham Heart Study began in 1948 to study the risk factors and characteristics of cardiovascular disease. Adults between the ages of 28 and 62 years and living in Framingham, Massachusetts were recruited for the study. A total of 5209 people participated in the baseline examination and have been examined every two years since. Among these participants, several were identified as being biologically related or spouse pairs.

To expand on the familial aspect of the study, the Framingham Offspring Cohort consisting of 3548 adult children of the original cohort and 1576 of their spouses were identified and recruited in 1971. This cohort has been followed roughly every four years (there were 8 years between the baseline and first follow-up exam). The protocols used were similar to the original Heart Study Cohort [10].

The family relationships available within each cohort were limited. In the original cohort there were a number of sibling and spousal pairs, but very few multi-generational relationships (parent-child for example). Also, there were very few cousin relationships that had been identified. In the offspring cohort, again there were only sibling and spousal pairs available. Combining the cohorts provided the full range of family relationships. The family pedigrees were identified in the 1980s and 330 of the largest pedigrees have been provided in this data set. This consists of 3041 parent-offspring pairs, 2796 sibling pairs, 2107 avuncular pairs, 183 grandparent-grandchild pairs, and 1595 first-cousin pairs.

For these analyses, we were interested in the continuous measurements that define the multiple metabolic syndrome. The relevant measures provided in the data set were: height and weight, HDL cholesterol and systolic blood pressure. Plasma triglycerides and glucose are important components of the multiple metabolic syndrome, but due to differences in collection between the cohorts, we only considered these measurements in the offspring cohort. Although both diastolic and systolic blood pressure would be of interest, only systolic was provided in the data set. Height and weight were not used individually, but were combined into body mass index (BMI) as a measure of obesity. BMI was calculated as weight (kg) divided by the square of the height (meters). We also used plasma total cholesterol since the HDL cholesterol was only measured at a few examinations.

Each cohort had their own follow-up cycle and the measurements collected varied somewhat at each exam. The original cohort was first examined in 1948 and has had follow-up every 2 years. The blood samples were from non-fasting venous samples, so any measure of glucose or triglycerides provided will not be considered for analyses. Blood pressure and weight were available at each follow-up exam. Plasma total cholesterol was available at most examinations. Height was available consistently after 1972. HDL cholesterol was only provided for the 11^th^, 15^th^, and 20^th ^exams (which occurred in 1968, 1976, and 1986). The offspring cohort was first measured in 1971–1975 then again in 1979–1982 and every 4 years after that. The blood samples were 12-hour fasting samples. All factors of interest for this analysis were measured at each examination phase. Unknown differences in exam techniques between the cohorts may affect the multi-generational correlations.

We selected examinations at a few points in time to examine correlations between pairs of relatives. We analyzed sibling correlations within each cohort separately for each of these times. We combined data from the two cohorts to maximally use the family relationships. Each examination phase for the offspring cohort encompassed two examination phases of the original cohort. In order to combine the cohorts, we averaged all available measurements for each cohort during specified calendar years. For example, from 1971 to 1975 the offspring cohort was being seen for their first exam. In 1972 the original cohort was being seen for their 13^th ^exam and in 1974 they were being seen for their 14^th ^exam. To have one measurement for the original cohort during the years 1971 to 1975, we averaged all available measurements from the 13^th ^and 14^th ^exams.

The first time chosen was 1968. At this time, only data from the original cohort were available (Exam 11). BMI was not available at this examination. The second time chosen was from 1971 to 1975. As described previously, the original cohort had participated in exams 13 and 14, from which measurements were averaged, while the offspring cohort had participated in their first exam. All measurements except HDL cholesterol were available for both cohorts. The third time chosen was from 1984 to 1987. During this time period, the original cohort had participated in Exams 19 and 20, from which measurements were averaged, while the offspring cohort had participated in Exam 3. All measurements were available for both cohorts at this time.

Analyses were performed using FCOR (for correlations and their standard errors) in the S.A.G.E. package [11]. Prior to analyses, the outcomes were adjusted for age (linearly) and gender. The residuals from these models were then used to calculate correlations. This was done in SAS [12].

## Results

The mean of each measure and the mean age of participants at each examination by cohort is shown in Table 1. The original cohort was older than the offspring cohort. The mean values for each variable at each examination, including those not considered in this paper, showed overall increasing trends with time. As seen in Table 1, the mean total cholesterol increased from 222 to 230 in the original cohort from Exam 11 to Exam 14. It dropped back to 213, however, by Exam 20. The mean systolic blood pressure was about 136 in the early 1970s for the original cohort and increased to about 144 in the 1980s. In the offspring cohort, the mean value at exam 1 was lower than at Exam 3 for all measures. In addition, the offspring cohort had lower measures than the original cohort at corresponding exam years.

Considering each examination and cohort separately, only sibling correlations were available (Table 2). The correlations were not affected by age and gender adjustment (unadjusted correlations not shown). The correlations for total cholesterol, BMI, and systolic blood pressure were very similar, but appeared to decrease somewhat with time (and age). The correlations for total cholesterol in the offspring cohort were 0.26 and 0.24, while the correlations in the original cohort ranged from 0.25 from Exam 11 to 0.16 in Exam 20. The correlations for BMI and systolic blood pressure also appeared to decrease over time, but the offspring and original cohort seemed quite similar. The correlations for BMI were 0.24 at both exams in the offspring cohort and declined from 0.27 at Exam 13 in the original cohort to 0.18 in Exam 20. The correlations for systolic blood pressure were 0.19 and 0.18 in the offspring cohort and declined from 0.18 at Exam 11 of the original cohort to 0.10 at Exam 19. The correlation increased to 0.15 at Exam 20, however. It should be noted that the confidence intervals were quite wide in the original cohort, and got wider with each exam, so these trends over time may not be meaningful. The correlations for HDL cholesterol were a bit more variable and any trends were difficult to detect since this was not always measured.

Since the correlations seemed fairly consistent, we combined the data as described in the Methods section. This provided sufficient numbers of pairs to estimate the parent-child, avuncular, and cousin correlations (Table 3). For all measures, the sib-sib correlations were always significantly different from 0. There were few differences between brother-brother, sister-sister, and brother-sister correlations (data not shown). The parent-child correlations were often close to the sib-sib correlations. The avuncular correlations were higher than the cousin correlations, but both were no different from 0. Spousal correlations were often just barely significantly different from 0. The correlations from measurements in 1971–5 were similar to those from 1984–7, except for the spousal correlations.

## Discussion

These data were supportive of a genetic component for HDL cholesterol and possibly for total cholesterol, BMI, and systolic blood pressure. For a trait whose variation is attributable entirely to additive genetic effects, the magnitude of the sibling and parent-child correlations should be similar, avuncular about half, and cousin correlations half again. Our sibling correlations were very close to the parent-child correlations for total plasma cholesterol, HDL cholesterol, and BMI. The avuncular and cousin correlations did drop by half for HDL cholesterol, but not for the other measures. These correlations were smaller than the sibling and parent-child correlations, however, and the cousin correlations were generally smaller than the avuncular correlations.

For traits whose variation is attributable to genetic effects, spousal correlations should be 0. Many of the spousal correlations were significantly different from 0 and were similar in magnitude to the parent-child correlations. The spousal correlation for BMI in exams from 1984 to 1987 was nearly double the parent-child correlation. This was highly suggestive of an environmental influence on these traits. The spousal correlations were more variable between the exam phases, especially for systolic blood pressure and BMI. This may just be related to the smaller number of pairs available for spousal correlations. The environmental influence was also supported by higher sibling correlation than the parent-child correlation. These correlations may also have been affected by even small differences in examination procedures. The majority of the spousal pairs were all from the original cohort, while the sibling pairs included some pairs from the original cohort and some from the offspring cohort. The parent-child and avuncular correlations were all between pairs where one individual was in the original cohort and the other individual was in the offspring cohort.

We observed a possible trend in decreasing correlations with time when considering the sibling correlations alone. In addition, we found the offspring cohort to have higher correlations than the original cohort, after adjusting for age. The decrease in correlation was very small, and likely not significant due to the wide confidence intervals. The decrease could be due to increasing variability in these measures that come with age or selective survival. Differences in morbidities within sibling pairs, causing a sibling to drop out of analyses, could affect the correlations. The spousal correlations were rather constant over time for plasma cholesterol and increased for BMI and systolic blood pressure.

## Conclusions

Family correlations of components of the multiple metabolic syndrome in the Framingham Heart Study and Offspring Study were consistent across outcomes and across cohorts. This was supportive of a genetic model to explain levels of these component characteristics. Segregation and linkage analyses may further our understanding of the genetic nature of each component. The clustering of these components has been shown to be predictive of cardiovascular disease as well as diabetes. Genes common to each of the components might also be responsible for these complex diseases.

## Figures and Tables

**Table 1:** Mean of measures at each exam and cohort.

				**Mean**
				
Year	Cohort	Exam #	N	Age	Plasma Total Cholesterol	HDL Cholesterol	Systolic Blood Pressure	Body Mass Index	Fasting Plasma Glucose	Fasting Plasma Triglycerides
1968	Original	11	1042	60.3	221.7	51.5	138.4	NA	NA	NA
1972	Original	13	972	63.9	229.7	NA	136.1	27.5	NA	NA
1974	Original	14	933	65.6	230.0	NA	134.3	27.4	NA	NA
1971–5	Offspring	1	1672	32.7	190.9	50.4	120.5	25.4	100.7	91.6
1984	Original	19	563	73.8	NA	NA	141.9	27.5	NA	NA
1986	Original	20	525	75.7	213.4	46.9	145.1	27.7	NA	NA
1984–7	Offspring	3	1407	45.3	206.9	50.8	121.0	26.7	93.1	122.4

**Table 2:** Sib-sib correlations (number of pairs) for each cohort/exam analyzed separately.

	1968		1971–1975			1984–1987	
	
Component	Original Cohort Exam 11	Original Cohort Exam 13	Original Cohort Exam 14	Offspring Cohort Exam 1	Original Cohort Exam 19	Offspring Cohort Exam 20	Offspring Cohort Exam 3
Plasma Total Cholesterol	0.25 (192)	0.25 (262)	0.19 (255)	0.26 (2112)		0.16 (93)	0.24 (1441)
HDL Cholesterol	0.33 (192)			0.20 (2086)		0.17 (93)	0.29 (1434)
Systolic Blood Pressure	0.18 (277)	0.13 (285)	0.12 (264)	0.19 (2156)	0.10 (119)	0.15 (102)	0.18 (1543)
Body Mass Index		0.27 (284)	0.26 (263)	0.24 (2162)	0.21 (113)	0.18 (87)	0.24 (1477)
Fasting Plasma Glucose				0.12 (2021)			0.09 (1437)
Fasting Plasma Triglycerides				0.13 (2109)			0.16 (1441)

**Table 3:** Familial correlations from combined data.

		1971 – 1975			1984–1987		
Component	Relationship	*N *(pairs)	Corr.	95% CI	*N *(pairs)	Corr.	95% CI

Plasma Total Cholesterol	Sibling	2522	0.26	(0.21, 0.32)	1595	0.25	(0.18, 0.32)
	Parent-Child	2225	0.21	(0.16, 0.27)	1005	0.14	(0.06, 0.21)
	Avuncular	1527	0.05	(-0.02, 0.13)	676	0.00	(-0.09, 0.10)
	Cousin	1566	0.03	(-0.06, 0.11)	948	-0.04	(-0.13, 0.05)
	Spousal	302	0.19	(0.08, 0.30)	127	0.13	(-0.04, 0.30)
HDL Cholesterol	Sibling				1588	0.27	(0.20, 0.34)
	Parent-Child				999	0.24	(0.16, 0.31)
	Avuncular				671	0.13	(0.02, 0.23)
	Cousin				940	0.06	(-0.04, 0.17)
	Spousal				126	0.07	(-0.11, 0.25)
Systolic Blood Pressure	Sibling	2576	0.18	(0.13, 0.24)	1756	0.17	(0.11, 0.23)
	Parent-Child	2278	0.07	(0.02, 0.12)	1287	0.06	(-0.01, 0.12)
	Avuncular	1573	0.04	(-0.02, 0.10)	857	0.08	(0.00, 0.16)
	Cousin	1591	-0.05	(-0.12, 0.01)	1017	-0.05	(-0.12, 0.03)
	Spousal	306	0.07	(-0.04, 0.19)	167	0.18	(0.03, 0.33)
Body Mass Index	Sibling	2580	0.25	(0.19, 0.31)	1675	0.24	(0.18, 0.30)
	Parent-Child	2274	0.21	(0.16, 0.26)	1196	0.17	(0.10, 0.24)
	Avuncular	1574	0.01	(-0.06, 0.08)	808	0.03	(-0.06, 0.12)
	Cousin	1598	-0.03	(-0.11, 0.04)	963	0.01	(-0.08, 0.10)
	Spousal	303	0.16	(0.05, 0.27)	160	0.34	(0.20, 0.47)
